# An endoscopic dilation method using the rendezvous approach for the treatment of severe anastomotic stenosis after rectal cancer surgery: a case report

**DOI:** 10.1186/s12957-020-02062-9

**Published:** 2020-11-07

**Authors:** Takuya Nakashima, Nobuhisa Matsuhashi, Tomonari Suetsugu, Yoshinori Iwata, Shigeru Kiyama, Takao Takahashi, Fukada Masahiro, Itaru Yasufuku, Yuta Sato, Takeharu Imai, Yoshihiro Tanaka, Naoki Okumura, Masaya Kubota, Takashi Ibuka, Masato Shimizu, Kazuhiro Yoshida

**Affiliations:** 1 Department of Surgical Oncology, Gifu University School of Medicine, Yanagido, Gifu City, 501-1194 Japan; 2 General and Cardiothoracic Surgery, Gifu University School of Medicine, Gifu City, Japan; 3 Department of Gastroenterology, Gifu University School of Medicine, Gifu City, Japan

**Keywords:** Colorectal cancer, Anastomotic stenosis, Endoscopic dilation

## Abstract

**Background:**

Postoperative anastomotic stenosis is a common complication in colorectal cancer patients (3–30%). Complete anastomotic stenosis is rare; however, when it occurs, almost all cases require surgical treatment. We herein report a case in which endoscopic dilation was effective for treating complete anastomotic stenosis after high anterior resection in a rectal cancer patient.

**Case presentation:**

The patient was a 67-year-old man who underwent laparoscopic high anterior resection for rectal cancer (RS, T4a, N0, M0, Stage IIB (TNM Classification of Malignant Tumors)) in May 2018. The postoperative course was good and the patient was discharged on the 12th postoperative day. Subsequently adjuvant chemotherapy was initiated with oral uracil and tegafur plus leucovorin (UFT/LV); however, he complained of frequent defecation and melena after completion of the first course of chemotherapy. Thus, colonoscopy was performed, which revealed anastomotic stenosis. Endoscopic dilation was initially attempted, but failed. Thus, low anterior resection was performed with diverting colostomy. Four additional courses of chemotherapy were administered for 1 month after surgery. At 6 months after the second surgery, colonoscopy was performed, and complete anastomotic stenosis was pointed out again. The patient was successfully treated by endoscopic dilation using the rendezvous method. After this treatment, the lumen of the anastomotic site was observed to have narrowed again and endoscopic dilatation to treat anastomotic stenosis was repeated. In addition, he received local injection of steroids in anastomotic stenosis site. The lumen of anastomotic stenosis remained after the local injection of steroids and closure of colostomy was performed 9 months after the second operation.

**Conclusions:**

Endoscopic dilation using the rendezvous method was effective for treating anastomotic stenosis after colorectal surgery.

## Background

The incidence of postoperative anastomotic stenosis in colorectal cancer patients ranges from 3 to 30% [1]. The causes of anastomotic stenosis include a history of irradiation, de-functionalization, anastomotic leakage and sepsis, and preoperative obesity [1]. Severe anastomotic stenosis, such that the lumen cannot be confirmed, is extremely rare. Once it occurs, almost all cases require surgical treatment. In the present case, endoscopic dilation was effective for treating complete anastomotic stenosis after surgery for rectal cancer.

## Case presentation

### Clinical course

**Fig. 1:**
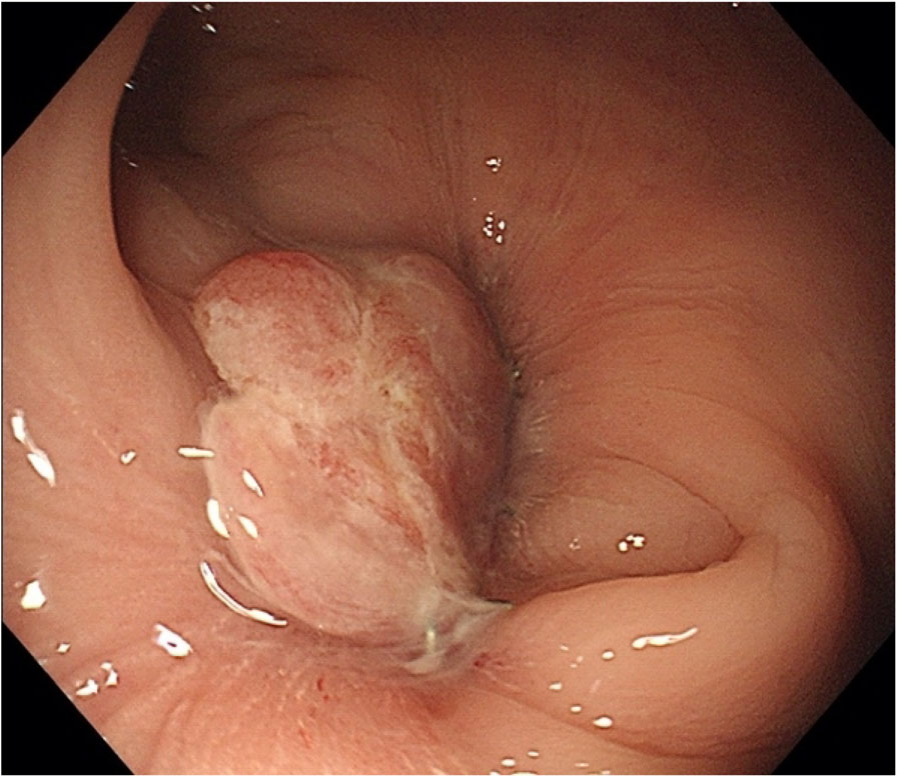
Severe anastomotic stenosis with granulation

The patient was a 67-year-old man who underwent laparoscopic high anterior resection with colorectal anastomosis using the double stapling technique (DST) as a treatment for rectal cancer (RS, T4a, N0, M0, Stage IIB (TNM Classification of Malignant Tumors)) in May 2018. The early postoperative course was uneventful and the patient was discharged on the 12th postoperative day without complication. Subsequently, adjuvant chemotherapy was started with oral uracil and tegafur plus leucovorin (UFT/LV); however, he complained of frequent defecation and melena after the completion of the first course of chemotherapy. Colonoscopy was therefore performed and anastomotic stenosis was pointed out. Granulation tissue development caused the stenosis to obstruct (Fig. 1). Biopsy of the granulated tissue showed no malignant findings. Endoscopic treatment was performed but failed due to intestinal perforation while searching for the lumen with the endoscopic balloon dilator and the guide wire. Low anterior resection was therefore performed with diverting colostomy as emergency surgery. After the operation, he was asymptomatic and completed five cycles of UFT/LV. At 6 months after the second operation, the patient indicated that he wanted the colostomy closed. Thus, colonoscopy was performed as a preoperative evaluation. Complete anastomotic stenosis was again pointed out. The length of the stenosis is about 10 mm. Due to concerns about strong adhesions of the pelvic cavity after two times surgeries, we planned endoscopic dilation using the rendezvous method to treat complete anastomotic stenosis.

### The procedure

**Fig. 2:**
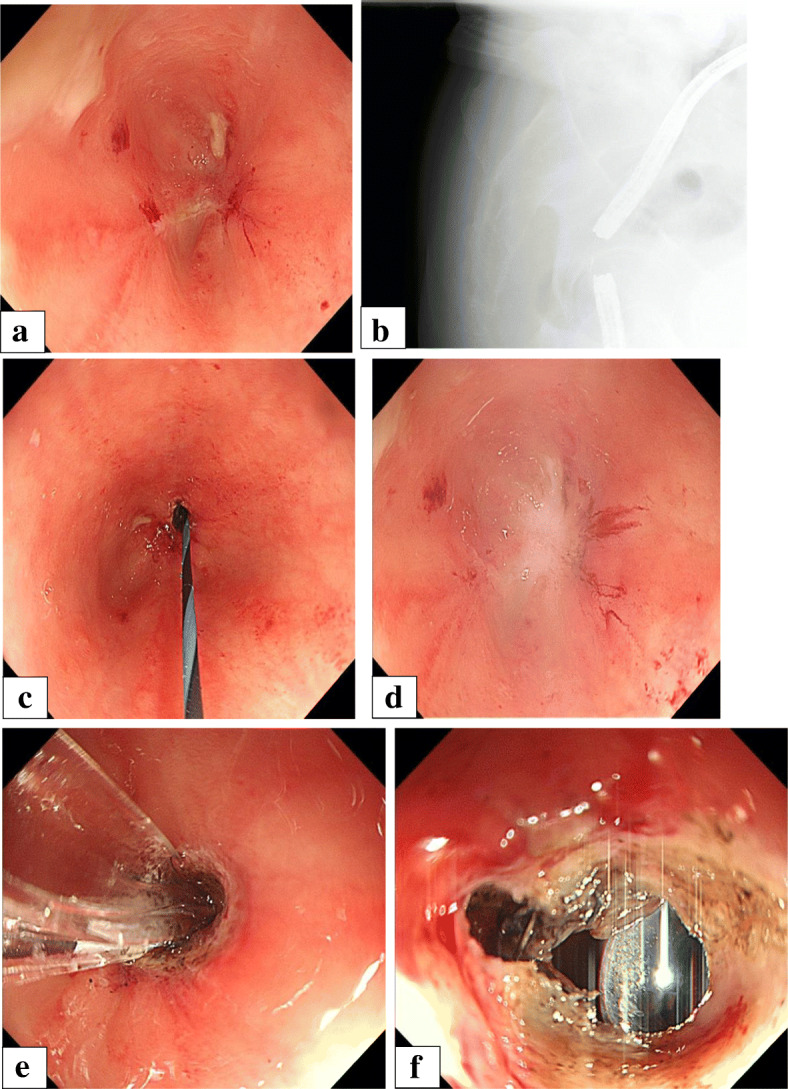
**a**–**f** Endoscopic dilation using rendezvous method. **a** Observed from oral side via colostomy and anal side, the anastomotic lesion became narrow, and the lumen of anastomosis was difficult to detect. **b** A radiographic image during rendezvous method. Endoscopes were inserted simultaneously from both the oral side via colostomy and anal side. The length of the stenosis is about 10 mm, and it is bent. **c**, **d** An incision was made from the anal side with a needle scalpel while looking at the light source from oral side via colostomy and a fluoroscopic image. However, because it did not open with that, next incision was made from oral side via colostomy and open it. **e** Dilation was performed from the anal side using 8.5–10.5-mm balloons at 1 atm, 2 atm, and 3 atm for 1 min each. **f** The stenosis was improved remarkably

**Fig. 3:**
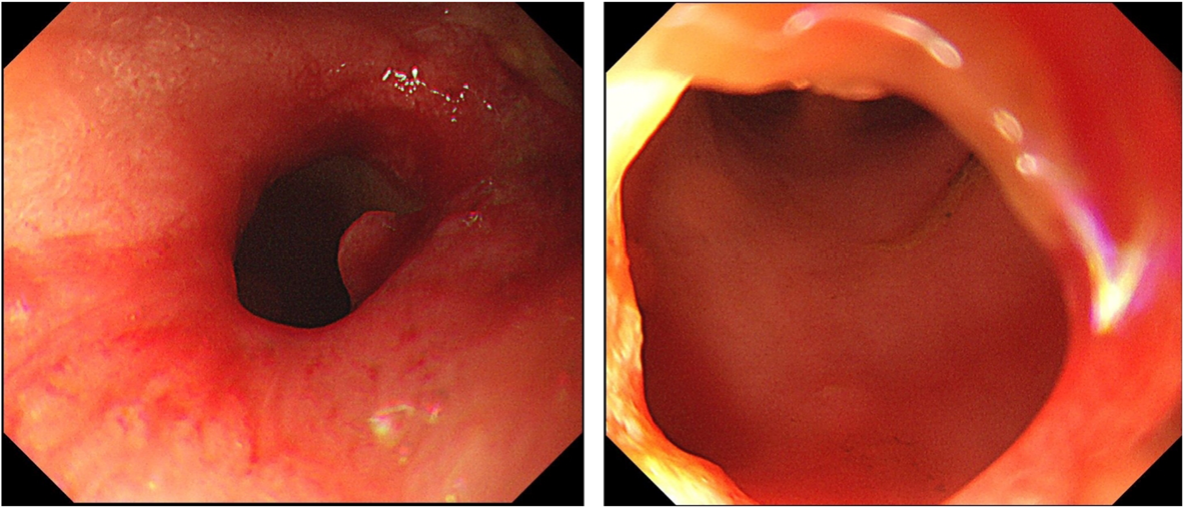
The stenosis was improved remarkably. Endoscopic image 9 months after the operation of stoma closure

The anastomotic lesion became narrow, and the lumen of anastomotic site was difficult to detect. Endoscopes (OLYMPUS GIF TYPE Q260) were inserted simultaneously from both oral side via colostomy and anal sides, and treatment was started (Fig. 2a–f). An incision was made from the anal side with a needle scalpel (needle scalpel, OLYMPUS) while looking at the light source from oral side via colostomy and a fluoroscopic image. However it did not open with that, so next incision was made from oral side via colostomy and open it. Dilation was subsequently performed from the anal side using 8.5–10.5-mm balloons (OLYMPUS EZDilate Endoscopic Balloon Dilator) at 1 atm, 2 atm, and 3 atm, for 1 min each. Finally, the stenosis showed remarkable improvement. No clear perforation was observed on endoscopy. After the treatment, the lumen of the anastomotic site was observed to have narrowed again and endoscopic dilatation was repeated. In addition, local injection of steroids (triamcinolone, 40 mg) was performed after endoscopic dilatation. The lumen of the anastomotic stenosis remained after the local injection of steroids and closure of the colostomy was performed 9 months after the second operation. A total of 11 times of endoscopic dilations were performed from endoscopic dilation using the rendezvous method to the stoma closure operation (Fig. 3a, b). He was discharged 30 days after surgery without problems with his defecation function or anastomotic stenosis.

**Fig. 4:**
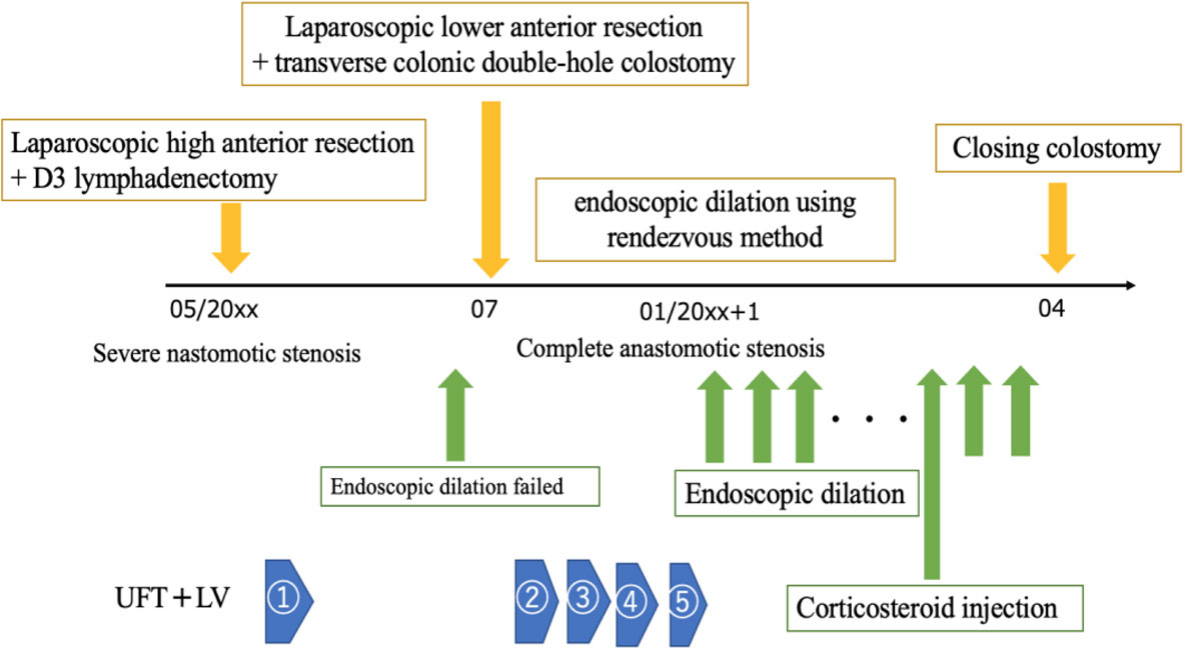
Clinical course. The equipment used for endoscopic dilation using rendezvous approach

Our case considered that this endoscopic procedure is useful as a method to release the complete obstruction of the anastomosis. In addition, local injection of steroid may be useful for the treatment of anastomotic stricture. In the course of the patient after closing colostomy, we have dilatations and local steroid injections at the same time, two times in total. After last procedure, the patients have no recurrence signs more than 12 months (Fig. 4).

## Discussion and conclusion

Postoperative anastomotic stenosis is a common complication that occurs in 3–30% of patients with colorectal cancer [1]. However, severe anastomotic stenosis such that the lumen cannot be confirmed is extremely rare. Endoscopic treatment is often difficult in such cases. Causes of anastomotic stenosis include—but are not limited to—infection, obesity, history of irradiation, impaired blood flow, postoperative leakage, and use of a stapling device [1, 2].

In general, anastomotic stenosis is associated with symptoms that often include difficulty in bowel movement, abdominal distension, and anal pain [3]. However, in cases in which colostomy is present, as in this case, attention is required as there are no symptoms related to stenosis. Various treatments for severe anastomotic stenosis, such as transanal surgery (TAMIS) [4] and endoscopic treatment with EUS [5], have been reported. In this case, repeating a transabdominal surgery, such as surgery of the pelvic cavity, are too invasive and risky and should only be considered as the last resort. Thus, less invasive options, such as balloon dilation, transanal stricturoplasty, stent placement, and stapler stricturoplasty, are preferable. We succeeded in treating complete anastomotic stenosis using the rendezvous method.

In 1987, Sommer et al. reported, for the first time, the principle of combining percutaneous and endoscopic approaches as the rendezvous technique [6]. The rendezvous technique is mainly used in the treatment of the biliary system [7]. Our search of the relevant literature in PubMed revealed that the rendezvous technique was first used for benign complete colonic anastomotic obstruction in 2006 [8]. There are a total of 11 cases of endoscopic dilatation using the rendezvous approach for the treatment of severe anastomotic stenosis after colorectal cancer surgery, and our case is the 12th case [5, 8–14] (Table 1). In the present case, colostomy was present; thus, we were able to treat the patient safely using the rendezvous method. A video of the surgery was reviewed after the occurrence of anastomotic stenosis; however, we could not identify any point within the surgical procedure that might have caused the anastomotic stenosis and anastomotic tension. Among the abovementioned risk factors, the possibility of impaired blood flow cannot be ruled out. Recently, the intravenous injection and monitoring ICG has been reported as a method for assessing anastomotic blood flow during surgery [15]. The local injection of steroids is also very effective for anastomotic stenosis. In this case, repeated endoscopic dilatation was performed even after the stenosis was released. However, local steroid injection successfully maintained the lumen of the anastomotic site after dilatation. Currently, this is the main method for treating anastomotic stenosis after ESD for esophageal cancer [16, 17]. Although there are few reports on the application of this method in the region of the colon and rectum [18], it seems to be a minimally invasive and effective option for treating anastomotic stenosis in such cases. In conclusion, endoscopic dilation using the rendezvous technique was an effective treatment for severe anastomotic stenosis. This procedure should be performed by an experienced endoscopist to avoid complications, such as intestinal perforation. This is therefore considered an effective method for the minimally invasive treatment of severe anastomotic stenosis.

**Table 1:** Cases of endoscopic dilation using the rendezvous approach for the treatment of severe anastomotic stenosis

	Author	Year	Age	Sex	Present illness	Stenosis site	Diverting stoma
1	Kaushik, N	2006	47	F	Sigmoid colon cancer	Sigmoid	ileostomy
2	Dever, J	2009	36	M	Colonic perforation	unknown	colostomy
3	Grossman, EB	2011	50	M	Rectal cancer	Rectum	cecostomy fistula
4	Albertsmeier,M	2011	73	M	Rectal cancer	Rectum	ileostomy
5	Dario Raimondo, D	2013	65	M	Rectal cancer	Rectum	ileostomy
6	Saxena, P	2015	54	M	Ischemic colitis	Rectum	ileostomy
7	Poincloux,L	2016	unknown	unknown	Rectosigmoid cancer	Rectum	colostomy
8	Poincloux,L	2016	unknown	unknown	Rectosigmoid cancer	Rectum	ileostomy
9	Sanaei, O	2017	44	F	Uterine leiomyoma	Rectum	ileostomy
10	Sanaei, O	2018	51	M	Rectosigmoid cancer	Rectum	ileostomy
11	Umair M.	2020	44	M	Rectal cancer	Rectum	ileostomy
12	Our Case	2020	67	M	Rectosigmoid cancer	Rectum	colostomy

## Data Availability

All data generated or analyzed during this study are included in this published article.

## Abbreviations

EUS: Endoscopic ultrasound
TAMIS: Transanal minimally invasive surgery
ICG: Indocyanine green
ESD: Endoscopic submucosal dissection
